# Factors in the success of the subcutaneous central venous port catheter in 626 colorectal carcinoma patients: long-term follow-up results according to the treatment groups

**DOI:** 10.1186/1470-7330-15-S1-P6

**Published:** 2015-10-02

**Authors:** BK Aribas, T Uylar, MY Aksoy, I Turker, F Yildiz, R Tiken, I Akdulum

**Affiliations:** 1 Dr. Abdurrahman Yurtaslan Ankara Oncology Education and Research Hospital Ankara, Turkey

## Aim

Our purpose is retrospectively to investigate the effect of factors on the patency of subcutaneous central venous port catheters inserted to 626 colorectal carcinoma patients.

## Patients and methods

Subcutaneous central venous port catheters were inserted to 1,408 patients. 241 were female, 383 were male. Patients were given chemotherapy agents as bevacizumab in 106, cetuximab in 30, and outside of these target-directed agents in 488. The groups to chemotherapy time were divided by 3 days cut-off. Age, gender, jugular-subclavian access, the chemotherapy to be given in 3 days as bevacizumab, cetuximab, other chemotherapy agents were also investigated.

## Results

The average age was 57.7 ± 11.3. The average follow-up period was 445.2 ± 387.8 days (1-1787 days). The catheters were removed depending on the port complications in 6 patients of bevacizumab group, 3 patients of cetuximab, and 11 patients of other chemotherapy group. A significant difference was observed in 3 days chemotherapy between cetuximab and out-of bevacizumab-cetuximab groups (p=0.013), also between bevacizumab and out-of bevacizumab-cetuximab the groups (p=0.007). No significant difference was observed out of 3 days chemotherapy (p>0.05). There was no difference between groups in Cox regression test. Significant difference was observed in catheter patency of bevacizumab-cetuximab group, due to the skin necrosis and thrombosis (p=0.011).

## Conclusion

Significant effect was found by reducing the duration time in patients with the target-directed chemotherapy agent as bevacizumab-cetuximab.

